# The COX-2 Selective Blocker Etodolac Inhibits TNFα-Induced Apoptosis in Isolated Rabbit Articular Chondrocytes

**DOI:** 10.3390/ijms141019705

**Published:** 2013-09-30

**Authors:** Kousuke Kumagai, Mitsuhiko Kubo, Shinji Imai, Futoshi Toyoda, Tsutomu Maeda, Noriaki Okumura, Hiroshi Matsuura, Yoshitaka Matsusue

**Affiliations:** 1 Department of Orthopaedic Surgery, Shiga University of Medical Science, Otsu, Shiga 520-2192, Japan; E-Mails: mkubo@belle.shiga-med.ac.jp (M.K.); simai@belle.shiga-med.ac.jp (S.I.); tsmaeda@belle.shiga-med.ac.jp (T.M.); noriaki@belle.shiga-med.ac.jp (N.O.); matsusue@belle.shiga-med.ac.jp (Y.M.); 2 Department of Physiology, Shiga University of Medical Science, Otsu, Shiga 520-2192, Japan; E-Mails: toyoda@belle.shiga-med.ac.jp (F.T.); matsuura@belle.shiga-med.ac.jp (H.M.)

**Keywords:** chondrocyte, apoptosis, TNFα, COX-2, osteoarthritis

## Abstract

Chondrocyte apoptosis contributes to the disruption of cartilage integrity in osteoarthritis (OA). Recently, we reported that activation of volume-sensitive Cl^−^ current (*I*_Cl,vol_) mediates cell shrinkage, triggering apoptosis in rabbit articular chondrocytes. A cyclooxygenase (COX) blocker is frequently used for the treatment of OA. In the present study, we examined *in vitro* effects of selective blockers of COX on the TNFα-induced activation of *I*_Cl,vol_ in rabbit chondrocytes using the patch-clamp technique. Exposure of isolated chondrocytes to TNFα resulted in an obvious increase in membrane Cl^−^ conductance. The TNFα-evoked Cl^−^ current exhibited electrophysiological and pharmacological properties similar to those of *I*_Cl,vol_. Pretreatment of cells with selective COX-2 blocker etodolac markedly inhibited *I*_Cl,vol_ activation by TNFα as well as subsequent apoptotic events such as apoptotic cell volume decrease (AVD) and elevation of caspase-3/7 activity. In contrast, a COX-1 blocker had no effect on the decrease in cell volume or the increase in caspase-3/7 activity induced by TNFα. Thus, the COX-2-selective blocker had an inhibitory effect on TNFα-induced apoptotic events, which suggests that this drug would have efficacy for the treatment of OA.

## 1. Introduction

Osteoarthritis (OA) is a progressive degenerative disease of the cartilage that leads to irreversible cartilage loss, joint pain and dysfunction. Chondrocyte apoptosis is a functionally important phenomenon in the development and growth of articular cartilage under physiological conditions. It is well known that in the processes of normal bone growth and endochondral ossification, terminally differentiated chondrocytes are removed from the calcified cartilage by apoptosis prior to the transition to bone [1]. Evidence is also accumulating that an increased incidence of chondrocyte apoptosis during aging is responsible for hypocellularity associated with degradation and/or pathological remodeling of the cartilage matrix, and exacerbates the risk of degenerative joint diseases such as OA [2–4]. In the cartilage of OA, disruption of the collagen network is accompanied by an increase in water content and a corresponding decrease in the pericellular osmolarity [5]. Articular chondrocytes are thus continuously exposed to perturbation of osmotic pressure and ionic composition.

The volume of articular chondrocytes rapidly decreases following the development of hyposmotic cell swelling (referred to as regulatory volume decrease, RVD), and several ion channels and transporters have been implicated in the volume regulation (for review, [6]). Our previous electrophysiological studies have shown that the volume-sensitive Cl^−^ current (*I*_Cl,vol_) is functionally expressed in rabbit articular chondrocytes and is involved in the RVD response after exposure to hyposmotic stress [7,8]. Recently, it is also suggested that aberrant activation of *I*_Cl,vol_ under isosmotic condition contributes to the cell shrinkage associated with induction of apoptosis (referred to as apoptotic volume decrease, AVD) in various cell types including chondrocytes [9]. In fact, various blockers of *I*_Cl,vol_ effectively prevent AVD and subsequent cell death induced by ischaemia-reperfusion stress or apoptotic inducers such as staurosporine, doxorubicin, Fas ligand, TNFα or sphingolipids [10].

Prostaglandins are important mediators that play a variety of roles in biological events and are known to be produced from membrane phospholipids by the sequential actions of phospholipase A2 and cyclooxygenase (COX). In particular, prostaglandin E2 is produced in bone mainly by osteoblasts, and it stimulates bone resorption [11–13]. There are two kinds of COX: a constitutive form, COX-1, that mediates physiological functions and an inducible form, COX-2, associated with pathological conditions such as inflammation [14–17]. Etodolac is an anti-inflammatory agent that potently and selectively inhibits COX-2 while preserving COX-1 activity [18], and is widely used to alleviate symptoms associated with OA [19]. Recently, accumulating evidence suggests that COX-2 regulates cell growth and proliferation as well as cell death in various tumors [20–22]. It is however still poorly understood whether COX-2 blocker affects chondrocyte apoptosis. In the present study, we examined *in vitro* effects of etodolac on TNFα-induced activation of *I*_Cl,vol_ in rabbit chondrocytes using the whole-cell patch-clamp technique. Our results show that etodolac effectively reverses TNFα-induced elicitation of *I*_Cl,vol_ and consequently inhibits AVD and elevation of caspase activity.

## 2. Results and Discussion

### 2.1. Expression of COX-1 and COX-2 in Rabbit Articular Chondrocytes

**Figure 1:**
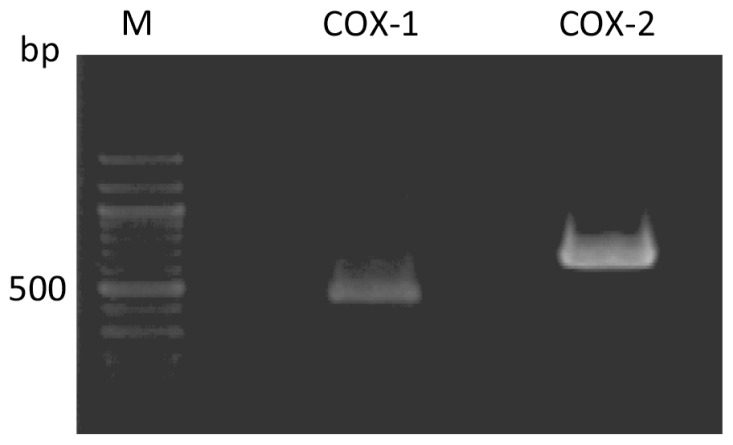
RT-PCR analysis of the expression of COX-1 and COX-2 in rabbit articular chondrocytes. PCR products obtained from freshly isolated chondrocytes were separated on a 2% agarose gel. Lane M is a size marker.

We first examined the expression of COX-1 and COX-2 in chondrocytes freshly isolated from cartilage (noncultured cells) by using RT-PCR analysis (Figure 1). In freshly isolated chondrocytes, bands corresponding to COX-1 (473 bp) and COX-2 (665 bp) were clearly observed.

### 2.2. TNFα-Induced Activation of Cl^−^ Current in Rabbit Articular Chondrocytes

**Figure 2:**
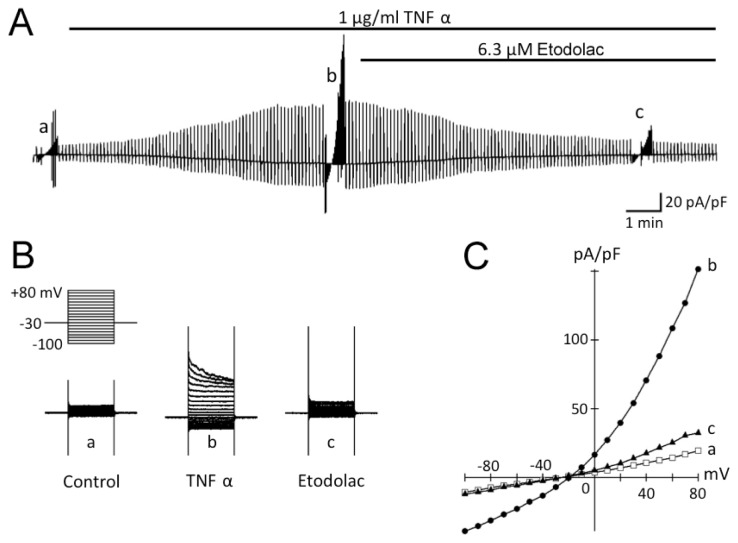
Activation of *I*_Cl,vol_ by an apoptosis inducer, TNFα. (**A**) Chart recording of the whole-cell current in response to voltage ramps (d*V*/d*t* = ±0.25 V/s, applied every 6 s) before and during application of TNFα; (**B**) The TNFα-evoked Cl^−^ current exhibited a prominent inactivation at larger positive potential than +50 mV; (**C**) An outward rectification of the *I–V* relationship with a reversal potential close to the Nernst *E*_Cl_ (−18.4 mV).

Figure 2 shows a representative experiment examining the effect of bath application of TNFα (1 μg/mL) on membrane currents in rabbit articular chondrocytes. Whole-cell currents were recorded under conditions designed to minimize Na^+^, K^+^ and Ca^2+^ currents and electrogenic Na^+^/K^+^ pump current. The Gd^3+^-sensitive stretch-activated channels were also blocked by adding 30 μM GdCl_3_ to the bath. During superfusion with control isosmotic solution, membrane currents elicited during square steps applied from a holding potential of −30 mV to test potentials between +80 and −100 mV were of small amplitude and practically time-independent (Figure 2A(a), B(a)). Bath application of 1 μg/mL TNFα under isosmotic conditions (360 mosmol/L) gradually activated the membrane current, which reached a steady level in about 10 min after drug application (Figure 2A). This TNFα-induced current, obtained by digital subtraction of membrane currents recorded before and during exposure to TNFα using the square-step protocol (Figure 2B), exhibited a marked inactivation at potentials positive to +50 mV (Figure 2B(b)) and an outward rectification with a reversal potential of −18.7 ± 0.3 mV (*n* = 5, *N* = 5; Figure 2C(b)), close to the equilibrium potential for Cl^−^ (*E*_Cl_ = −18.4 mV) under the present experimental conditions. This increase in membrane current was not accompanied by an appreciable change in cell size (a, diameter, 13.8 ± 0.2 μm; b, 13.8 ± 0.2 μm; *n* = 5, *N* = 5), as assessed by measuring the cross-sectional area of microscopic cell images.

We next examined the effect of bath application of etodolac (6.3 μM). *I*_Cl,vol_ was gradually but almost totally inhibited by subsequent application of etodolac (93.4 ± 3.6% inhibition by 10 min exposure; *n* = 5, *N* = 5; Figure 2A). The *I–V* relationship of membrane current inhibited by etodolac displayed an outward rectification and a reversal potential close to the Nernst *E*_Cl_ (−18.4 mV), suggesting that only *I*_Cl,vol_ was affected by etodolac.

### 2.3. TNFα-Induced AVD and Its Inhibition by Etodolac

**Figure 3:**
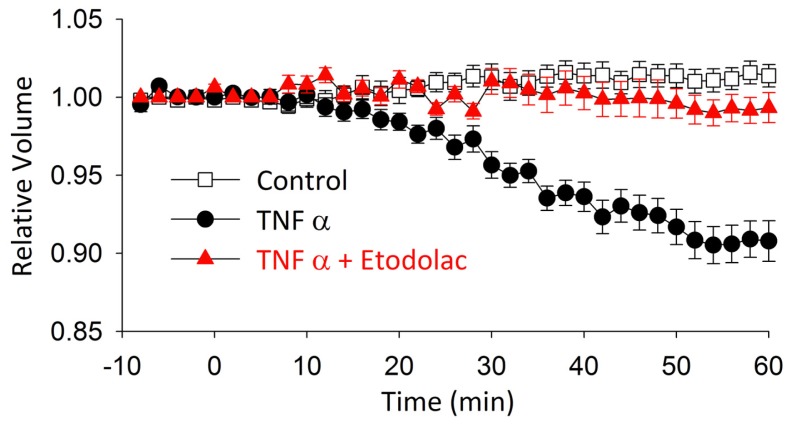
Effect of etodolac on TNF-α-induced decrease in relative cross-sectional area of cells. At time 0, drugs were added to the perfusion fluid.

It has been demonstrated in various cell types that exposure to TNFα induces AVD [23–25], which is mediated through activation of *I*_Cl,vol_[9]. To elucidate the functional significance of *I*_Cl,vol_ in articular chondrocytes, the effect of TNFα on cell size was examined in the absence and presence of etodolac, a compound that potently blocks *I*_Cl,vol_ (Figure 2A). The addition of TNFα to the isosmotic solution led to a gradual decrease in relative cell size (0.90 ± 0.01; *n* = 5, *N* = 5) over a period of 60 min in the presence of TNFα (Figure 3). Etodolac completely abolished the TNFα-induced decrease in cell size (0.99 ± 0.01; *n* = 5, *N* = 5), suggesting that *I*_Cl,vol_ is primarily involved in mediating the TNFα-induced decrease in cell size.

### 2.4. TNFα-Induced Caspase 3/7 Activity

**Figure 4:**
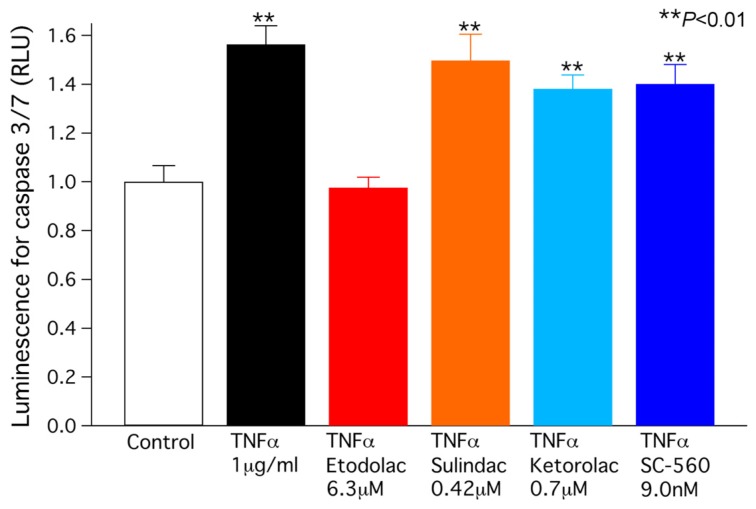
Caspase-3/7 activity, measured in chondrocytes after a 48-h exposure to TNF-α (1 μg/mL) without or with the COX-2 inhibitor etodolac and the COX-1 inhibitors sulindac, ketorolac and SC-560. Asterisks represent *p* values according to the Newman-Keuls multiple means comparison test (**p < 0.01).

A preliminary experiment in our laboratory demonstrated that the activity of caspase-3/7, a dominant effector for final apoptotic cell death [26,27], is appreciably elevated over a period of time (e.g., 48 h) after exposure to TNFα. To examine whether the cell size decrease induced by TNFα leads to apoptosis, caspase-3/7 activity was measured in chondrocytes exposed for 48 h to TNFα (1 μg/mL) without and with COX-1 or COX-2 inhibiters at the concentration relevant to clinical use. As expected, caspase-3/7 activity was markedly elevated by exposure to TNFα (Figure 4), indicating that the apoptotic signal was indeed evoked in chondrocytes by TNFα. This elevation of caspase-3/7 activity was completely abolished by the COX-2 inhibitor etodolac (6.3 μM), indicating that the activation of *I*_Cl,vol_ and the resulting decrease in cell size are essential for the elevation of caspase-3/7 activity. In contrast, the COX-1 inhibitors sulindac (0.42 μM, ketorolac (0.7 μM) and SC-560 (9.0 nM) had no effect on the increase in caspase 3/7 activity induced by TNFα.

TNFα evokes apoptosis in various cell types, as evidenced by AVD, the elevation of caspase activity and/or the induction of DNA fragmentation [28–30]. Our previous study have indicated that DCPIB (a selective *I*_Cl,vol_ blocker) prevents doxorubicin-induced AVD and caspase elevation in rabbit articular chondrocytes, suggesting that the activation of *I*_Cl,vol_ is a crucial step for the induction of chondrocyte apoptosis [9]. Consistent with this view, our present study revealed that *I*_Cl,vol_ was readily activated by TNFα even under isosmotic condition (Figure 2). It is assumed that the isosmotic activation of *I*_Cl,vol_ during chondrocyte exposure to TNFα mediates the efflux of intracellular osmolytes (such as K^+^ and Cl^−^) and osmotically obliged water leading to a AVD. In the present study, caspase-3/7 activity elevation was completely abolished by COX-2 inhibitor not but COX-1 blocker, suggesting that PGE2 is not mainly involved in the anti-apoptotic effect of COX-2 inhibitor. Alternatively, one of potential mechanisms for the differential effects of selective COX-1 and COX-2 inhibitors is via their efficacy for *I*_Cl,vol_ modulation. In our preliminary observations, COX-1 blocker failed to inhibit *I*_Cl,vol_ activation induced by TNFα in contrast to etodolac. Taken together, the present study provides a novel insight for the mechanism underlying the anti-apoptotic effect of COX-2 blocker. Because in the present experiments, chondrocyte apoptosis was induced by pharmacological (TNFα) interventions, the results cannot be directly extrapolated to chondrocyte apoptosis associated with OA in humans. Future studies are awaited to examine whether and how etodolac has a favorable action against OA chondrocyte in humans.

## 3. Experimental Section

### 3.1. Chondrocyte Isolation

All of the experimental protocols conform to The Guide for the Care and Use of Laboratory Animals [31] and were approved by the Animal Care and Use Committee of Shiga University of Medical Science. Articular chondrocytes were isolated from 15 adult male Japanese white rabbits (body weight, 2.5 to 3 kg) using an enzymatic dissociation procedure similar to that described previously [32] with modifications [8]. In brief, rabbits were deeply anaesthetized by intramuscular injection of ketamine (70 mg/kg) and xylazine (5 mg/kg) and then killed by intravenous injection of sodium pentobarbital (70 mg/kg). Articular cartilage was removed from bilateral knee, hip and shoulder joints and washed with phosphate-buffered saline (PBS; ICN Biomedicals Inc., Aurora, OH, USA). The sliced cartilage samples were incubated in plastic culture dishes containing Dulbecco’s modified Eagle’s medium (DMEM; Gibco BRL, Grand Island, NY, USA) supplemented with 10% fetal calf serum and antibiotics in a humidified atmosphere of 95% air plus 5% CO_2_ at 37 °C for 1 to 3 days. On the day of the experiments, the cartilage samples were cut into small pieces (~1 mm^3^) and digested with 0.5% collagenase (Type 2; Worthington Biochemical Corp., Lakewood, NJ, USA) for 4 h. Dispersed chondrocytes were washed three times, resuspended in DMEM supplemented 40 mM mannitol (~360 mosmol/L) and used for experiments within 8 h after isolation.

### 3.2. Solutions and Chemicals

The isosmotic external solution used for the patch-clamp experiments contained (in mM): mannitol 150, NaCl 100, MgCl_2_ 2.0, BaCl_2_ 2.0, glucose 5.5, and Hepes 10 (pH adjusted to 7.4 with NaOH). The osmolarity of these external solutions, measured with a freezing point depression osmometer (Fiske, Burlington, MA, USA), averages 360 mosmol/L. This high osmolarity is based on the fact that cartilage osmolarity is much higher than that of other tissue [33]. In addition, a previous study reported that the volume sensitive response in bovine chondrocytes is attenuated at medium osmolarity commonly used for most cell types (~280 mosmol/L) [34]. The standard pipette solution contained (in mM): aspartate 135, tetraethylammonium chloride 20, MgCl_2_ 2.0, Tris-ATP 5.0, Na_2_-GTP 0.1, EGTA 5.0, and Hepes 5.0 (pH adjusted to 7.2 with CsOH). The concentrations of free Ca^2+^ and Mg^2+^ in the pipette solution were calculated to be approximately 1.5 × 10^−10^ M (pCa = 9.8) and 5.1 × 10^−5^ M (pMg = 4.3), respectively [35,36]. The isosmotic external solution used for measuring AVD contained (in mM): mannitol 180, NaCl 90, KCl 5.4, CaCl_2_ 1.8, MgCl_2_ 0.5, NaH_2_PO_4_ 0.33, glucose 5.5, and Hepes 5.0 (pH adjusted to 7.4 with NaOH).

Test compounds were added to the isosmotic external solutions, as denoted by horizontal bars in the figures. These included TNFα (R & D Systems, Minneapolis, MN, USA) and etodolac (Wako, Osaka, Japan). The following stock solutions were made: 25 μg/mL TNFα in PBS with bovine serum albumin and 1 mM etodolac in ethanol. These were stored in aliquots at −20 °C.

### 3.3. Whole-Cell Patch-Clamp Technique and Data Analysis

Whole-cell membrane currents [37] were recorded from isolated chondrocytes by using an EPC-8 patch-clamp amplifier (HEKA, Lambrecht, Germany). Fire-polished pipettes pulled from borosilicate glass capillaries (Narishige Scientific Instrument Lab., Tokyo, Japan) had a resistance of 2.0 to 4.0 MΩ when filled with the standard pipette solution. Either square-step or voltage-ramp protocols were used to record the whole-cell current. Voltage ramps were used to monitor the time course of changes in membrane currents during various interventions, while the steady-state effects were recorded by using square voltage steps, unless otherwise stated. The voltage ramp protocol (d*V*/d*t* = ±0.25 V/s) was repeated every 6 s and consisted of three phases: an initial +80 mV depolarizing phase from a holding potential of −30 mV, a second hyperpolarizing phase of −150 mV, and then a third phase returning to the holding potential. The holding potential of −30 mV was used to avoid possible contamination of the voltage-gated Na^+^ current in our recordings. The current-voltage (*I*–*V*) relationship was measured during the second hyperpolarizing phase. Changes in the swelling-induced membrane conductance were evaluated from a linear least-squares fit to the *I*–*V* curves at potentials within an approximately 20 mV range centered on the reversal potential [38,39]. Voltage-clamp protocols and data acquisition were controlled with a Patchmaster software (v. 1.03; HEKA, Lambrecht, Germany), and current records were filtered at 1 kHz, digitized at 5 kHz through a LIH-1600 interface (HEKA), and stored on a Macintosh computer. Cell-membrane capacitance (*C*_m_) was calculated from the capacitive transients elicited by 20-ms voltage-clamp steps (±5 mV) from a holding potential of −30 mV, by using the following relationship [40]: *C*_m_ = τ_c_*I*_0_/Δ*V*_m_ (1 − *I*_∞_/*I*_0_), where τ_c_ is the time constant of the capacitive transient, *I*_0_ is the initial peak current amplitude, Δ*V*_m_ is the amplitude of the voltage step (5 mV), and *I*_∞_ is the steady-state current. The sampling rate for these measurements of *C*_m_ was 50 kHz with a low-pass 10 kHz filter. Membrane current amplitude and slope conductance were normalized to *C*_m_ for each cell and expressed as pA/pF and pS/pF, respectively. The zero-current level is indicated by arrowhead to the left of the current traces in the figures.

### 3.4. Microscopy and Image Analysis

An aliquot of cell (chondrocyte) suspension was transferred to a recording chamber (0.5 mL in volume) mounted on the stage of a Nikon eclipse TE2000-U inverted microscope (Tokyo, Japan) and at least 5 min was allowed for the cells to adhere lightly to the glass bottom. The chamber was continuously perfused at a constant rate of 2 mL/min with an external solution at 36 ± 1 °C, and the external solution was changed by switching the perfusates at the inlet of the chamber, with a complete change in bath solution taking 15 to 20 s. All cell size measurements and patch-clamp experiments were conducted on round-shaped healthy chondrocytes. Light microscopy images of chondrocytes were captured consecutively at 1 min intervals at a 2560 × 1920 pixel resolution by a CCD digital camera (DS-Fi1; Nikon, Tokyo, Japan) equipped with a DS-L2 control unit (Nikon, Tokyo, Japan). The cross-sectional area of each chondrocyte was measured by counting the pixels contained within the cell image, using Image-J public domain software (NIH, Bethesda, MD, USA). The value was converted into the cell volume simply by using the general formula and normalized to its initial isosmotic size obtained 1 min before switching to test solutions. The percentage AVD was calculated as follows: (peak volume − volume at test time)/(peak volume − 1) × 100, where the test time is 60 min.

### 3.5. Caspase-3/7 Activity Measurement

Caspase-3/7 activity was measured in chondrocytes treated with 1 μM TNFα for 48 h with or without various test compounds. Briefly, cells were lysed and the supernatant was collected for the measurement of caspase-3/7 activity by using the Caspase-Glo 3/7 assay system (Promega, Madison, WI, USA) according to the manufacturer’s instructions. The luminescent signal was measured with a luminometer (Infinite M200; Tecan, Mannedorf, Switzerland).

### 3.6. Statistical Analysis

Data values are expressed as means ± S.E.M., with the number of animals (cell isolations) and cells from which measurements were made indicated by *N* and *n*, respectively. Statistical comparisons were evaluated by using either Student’s *t* test or analysis of variance (ANOVA) followed by a *post hoc* Newman-Keuls test, and differences were considered significant at *p <* 0.05.

## 4. Conclusions

The COX-2 selective blocker etodolac had an inhibitory effect on TNFα-induced apoptotic events, which suggests that this drug would have efficacy for the treatment of chronic destructive joint disease.
